# Supplementary figures and images for: Common alleles of CMT2 and NRPE1 are major determinants of CHH methylation variation in Arabidopsis thaliana
Source: PLoS Genet. 2019 Dec 30;15(12):e1008492. doi: 10.1371/journal.pgen.1008492 (PMC6953882; doi:10.1371/journal.pgen.1008492)

S1 Fig

**A**

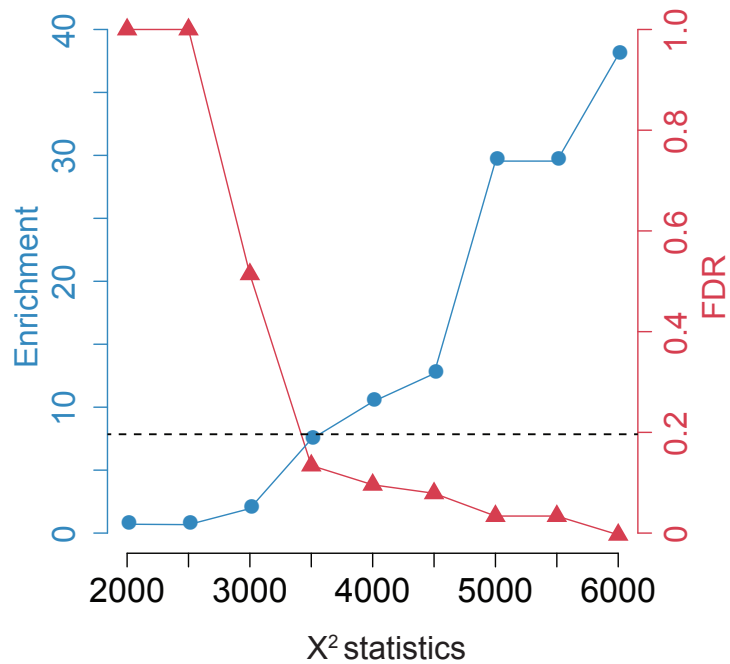

**B**

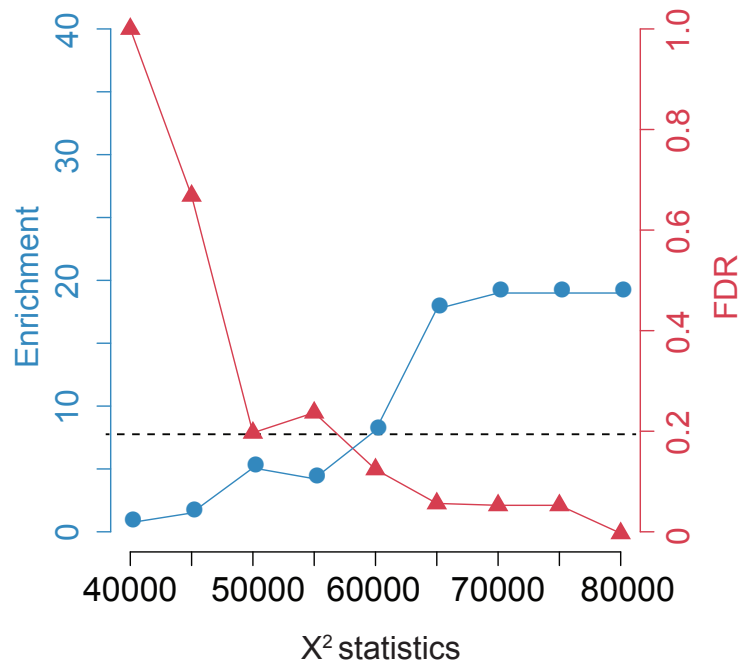

Supplement: S1 Fig — Enrichment and FDR 20% based on a priori genes (see Methods and also [12]). The horizontal dashed line at 0.2 corresponds to FDR 20%. (PDF) [file pgen.1008492.s001.pdf]

S2 Fig

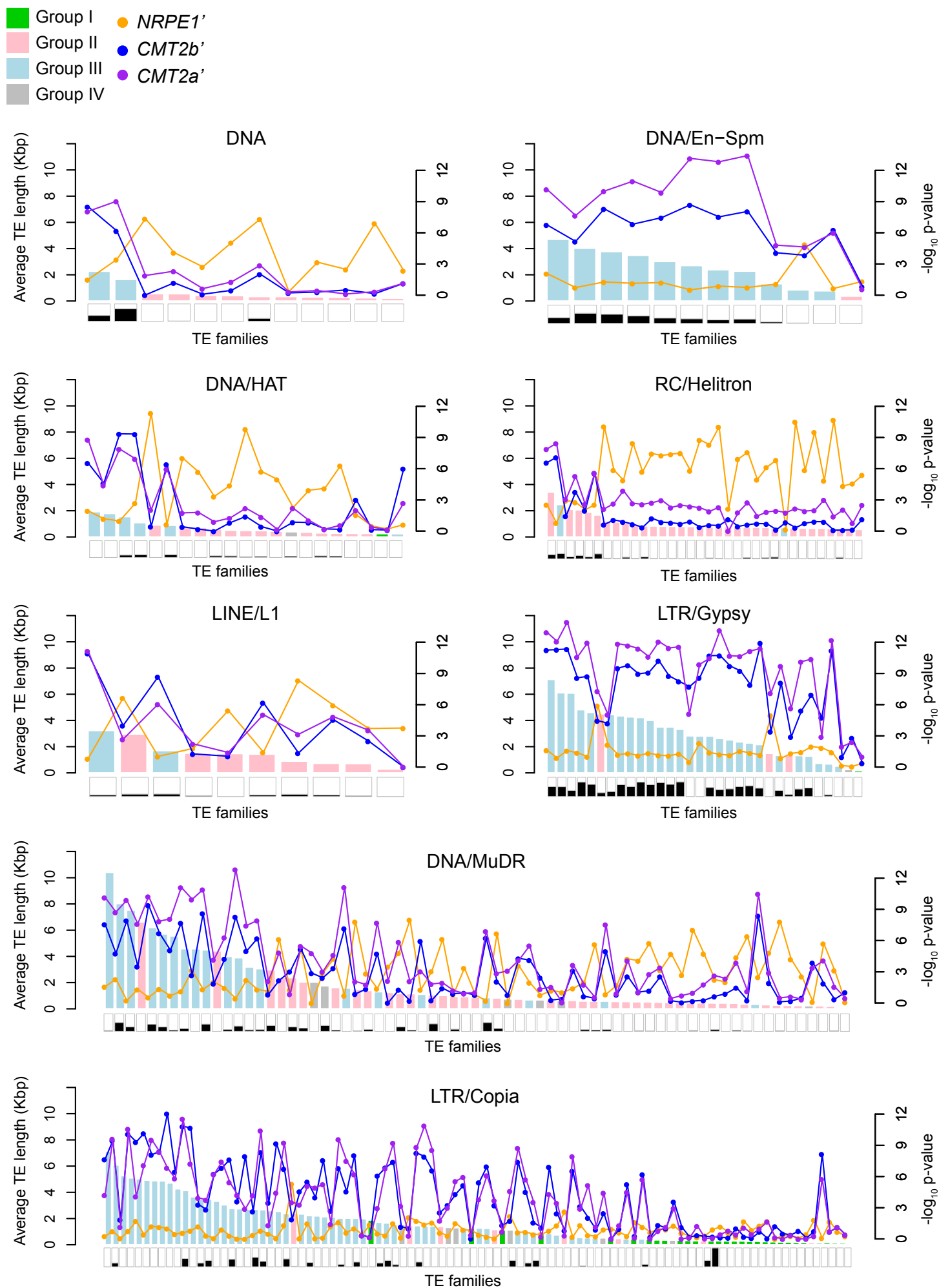

Supplement: S2 Fig — Bar plots indicate the average length of TE families ordered by the length with GWAS p-values for three alleles (line plots; see also S1 Table) and the proportion of TEs located around centromeric regions (black fraction in bar plots; 1Mbp from centromeric regions). (PDF) [file pgen.1008492.s002.pdf]

S3 Fig

**A**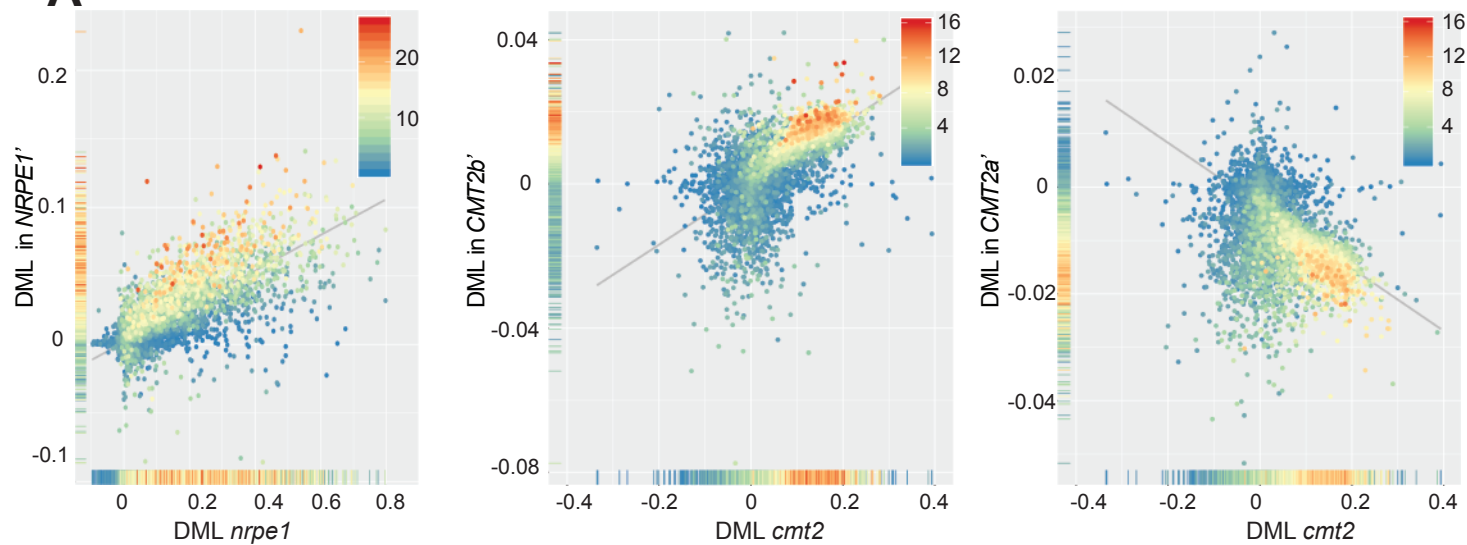**B**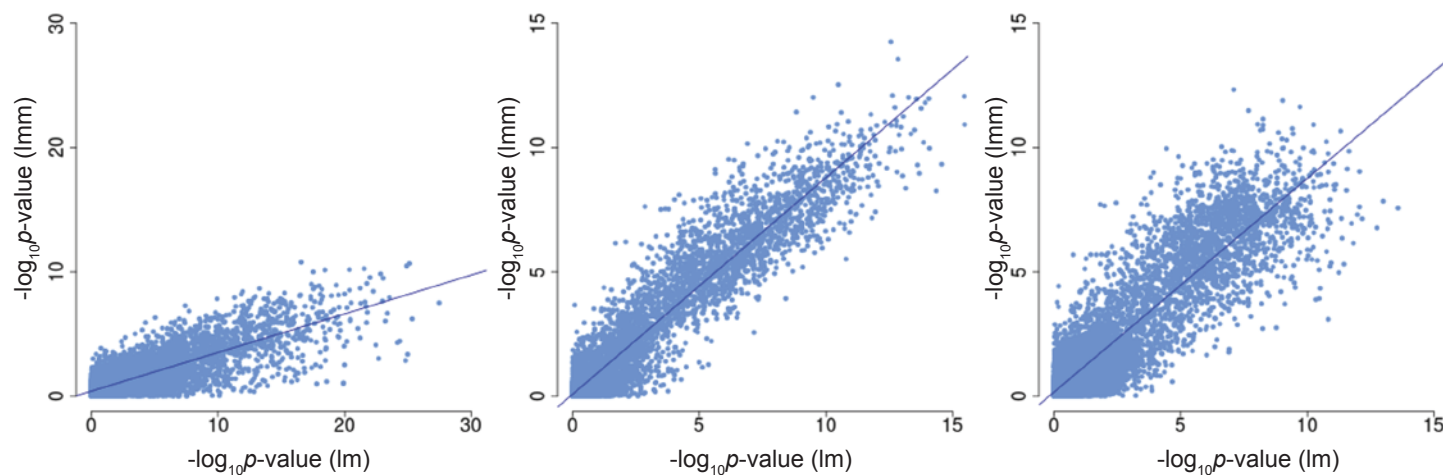

Supplement: S3 Fig — (A) Scatter plots show correlations of differential mCHH levels (DML) induced by alleles and mutants for each TE. DML for alleles was estimated as average differences of mCHH levels between lines carrying reference and non-reference alleles, whereas for mutants it was estimated between wild-type and nrpe1-11 or cmt2. Colors of dots in the scatter plots show the significance of the allelic effects as -log10p-value in GWAS (a linear model without correction of population structure). Density plots on Y and X-axis show distributions of the allelic effects for TEs. (B) Effects of population structure for mCHH levels of individual TEs. Scatter plots show -log10p-values estimated by a linear model (lm in X-axis) and a linear-mixed model (lmm in Y-axis). (PDF) [file pgen.1008492.s003.pdf]

S4 Fig

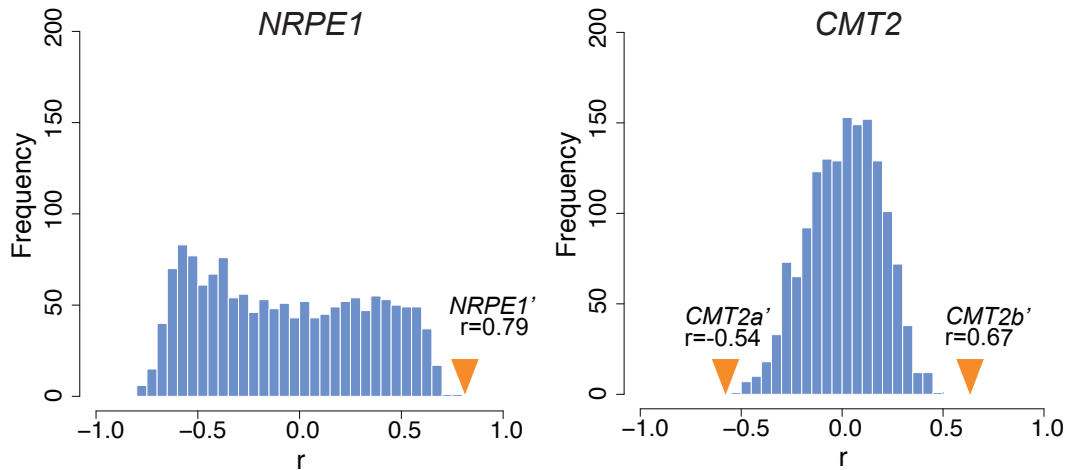

Supplement: S4 Fig — Spearman’s correlation coefficients (r) were calculated between DML of candidate mutants (nrpe1-11 and cmt2) and 1500 randomly picked up SNPs over the genome (see Methods). Orange arrows show r of NRPE1’, CMT2a’, and CMT2b’. All allelic effects were significantly stronger than randomly picked up SNPs (p<0.001). (PDF) [file pgen.1008492.s004.pdf]

# S5 Fig

- mutants in RdDM pathway
- mutants in CMT2 pathway
- Other mutants
- Candidates

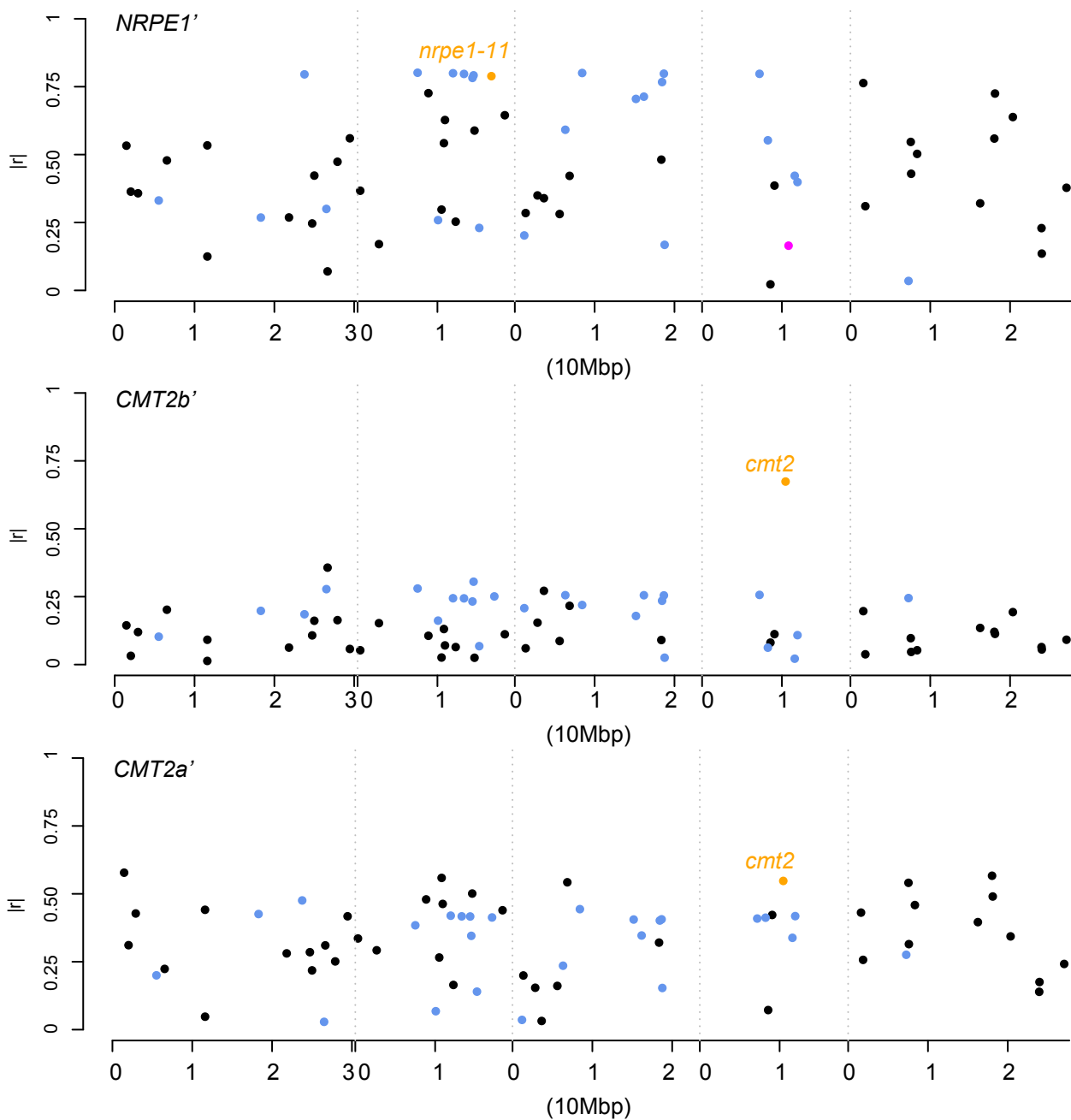

Supplement: S5 Fig — Each dot shows the absolute value of Spearman’s correlation coefficients r between DML of the three alleles and 67 single knockout mutants [13] along with the gene location on the genome. (PDF) [file pgen.1008492.s005.pdf]

S6 Fig

*NRPE1*

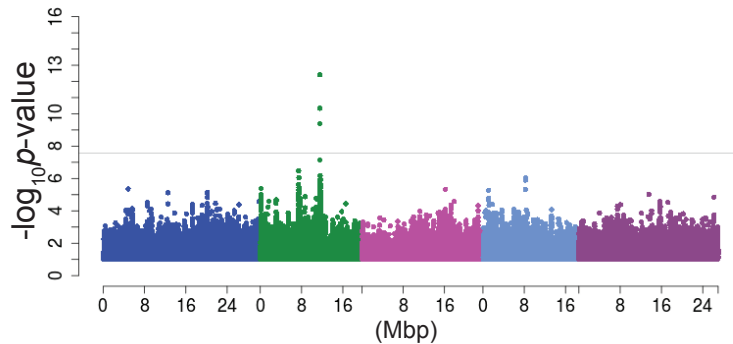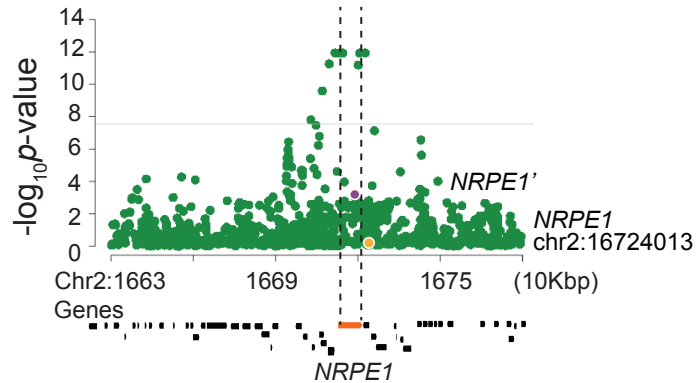

Supplement: S6 Fig — Manhattan plots and the cis peaks for NRPE1 expression (n = 665; leaf tissue under 21°C). Horizontal lines show the threshold (p-value 5% Bonferroni correction). (PDF) [file pgen.1008492.s006.pdf]

S7 Fig

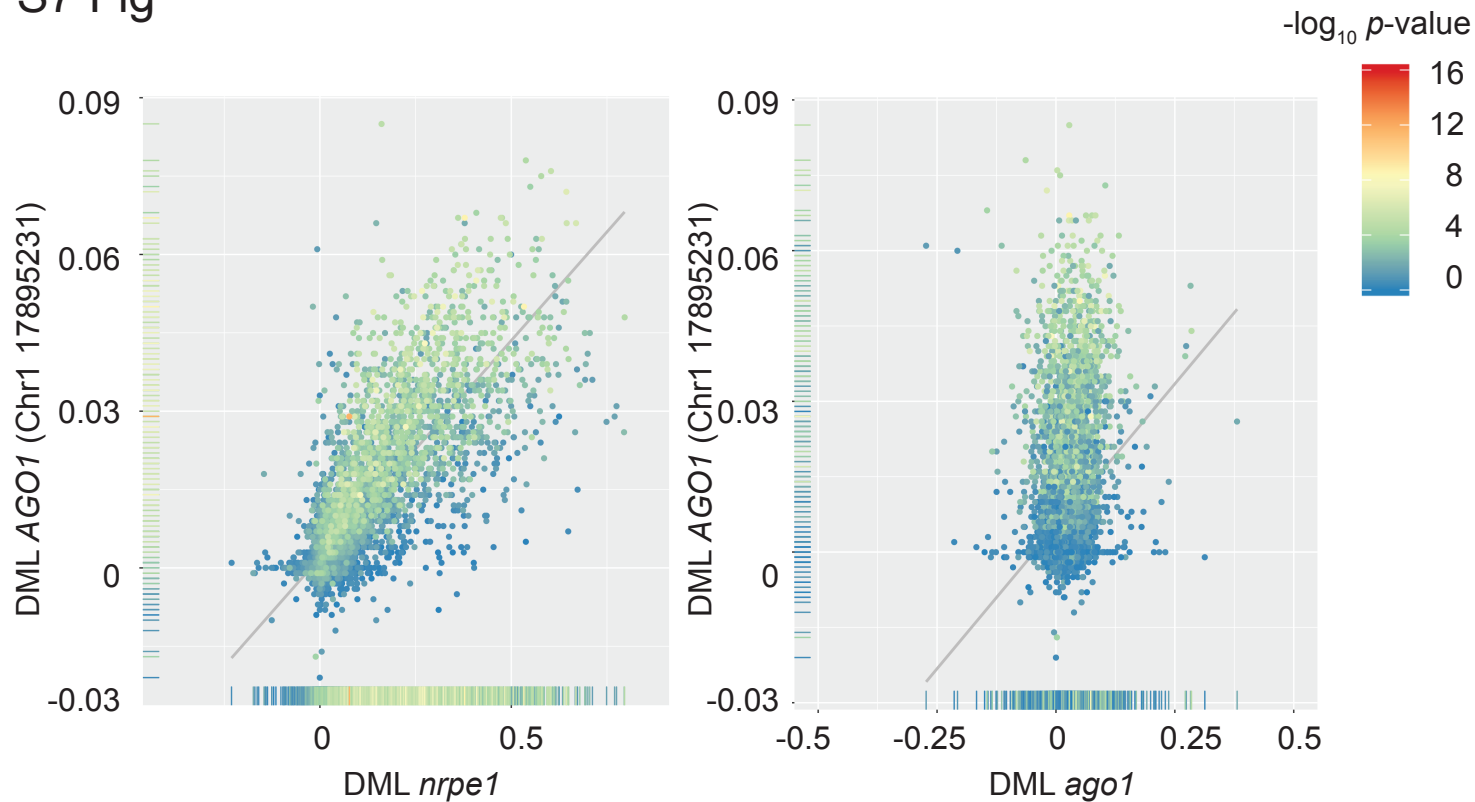

Supplement: S7 Fig — Scatter plots show correlations of DML induced by NRPE1’ and mutants, nrpe1-11 and ago1, for each TE. DML for alleles was estimated as average differences of mCHH levels between lines carrying reference and non-reference alleles, whereas it for mutants was estimated between wild-type and nrpe1-11 and ago1 loss-of-function. Colors of dots in the scatter plots show the significance of the allelic effects as -log10p-value in GWAS. Density plots on Y and X-axis show distributions of the allelic effects for TEs. (PDF) [file pgen.1008492.s007.pdf]

S8 Fig

**A**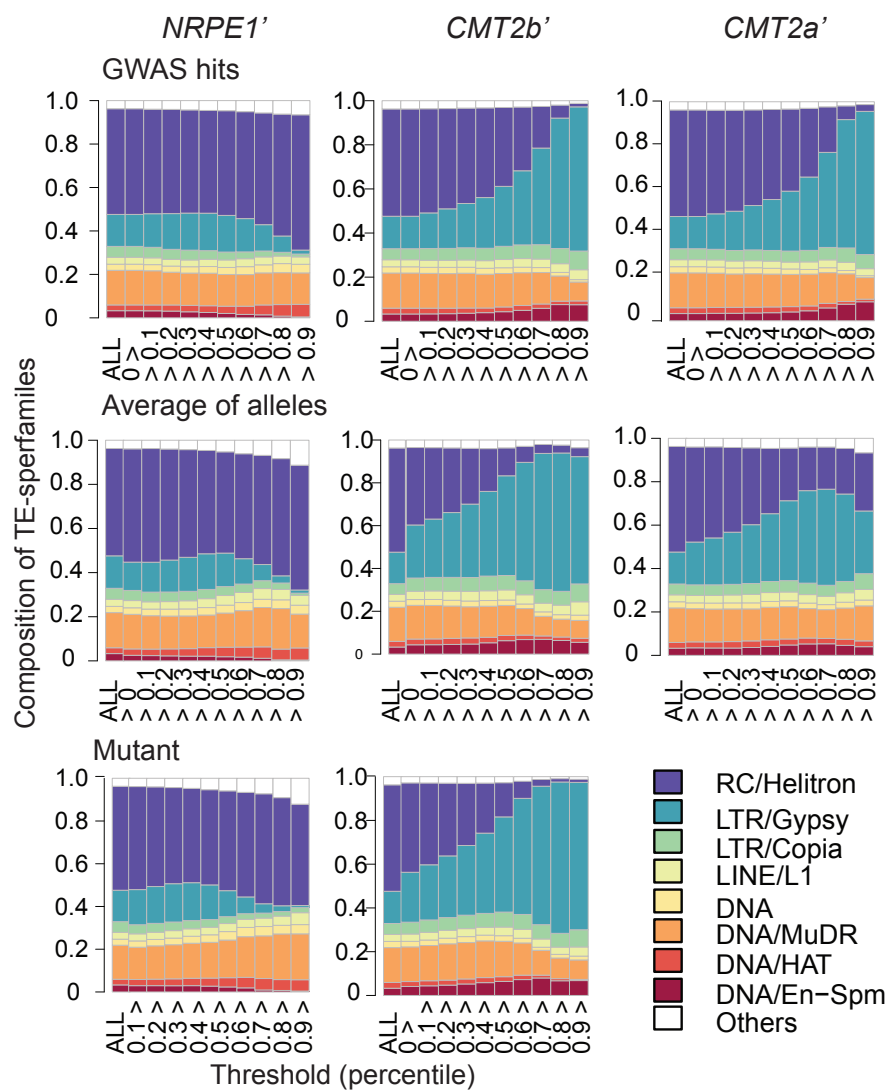**B**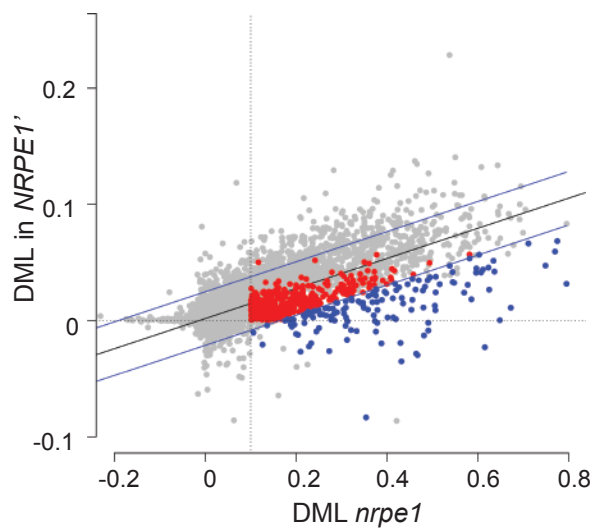**C**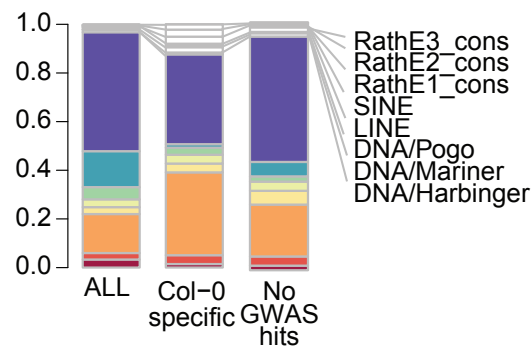

Supplement: S8 Fig — (A) Compositions of TE-superfamilies identified by GWAS, population-based average, or loss-of-function mutants at 0 to 90 percentile thresholds. (B) The scatter plot shows the correlation between DML induced by NRPE1’ and nrpe1 loss-of-function with 95% confident prediction. Blue dots indicate TEs showing nrpe1-1 loss-of-function specific effects on DML, and red dots indicate TEs that were not detected by GWAS regardless of the DML (lm -log10p-value > 3). (C) Composition of TE-superfamilies shown in panel B (blue and red dots). (PDF) [file pgen.1008492.s008.pdf]

S9 Fig

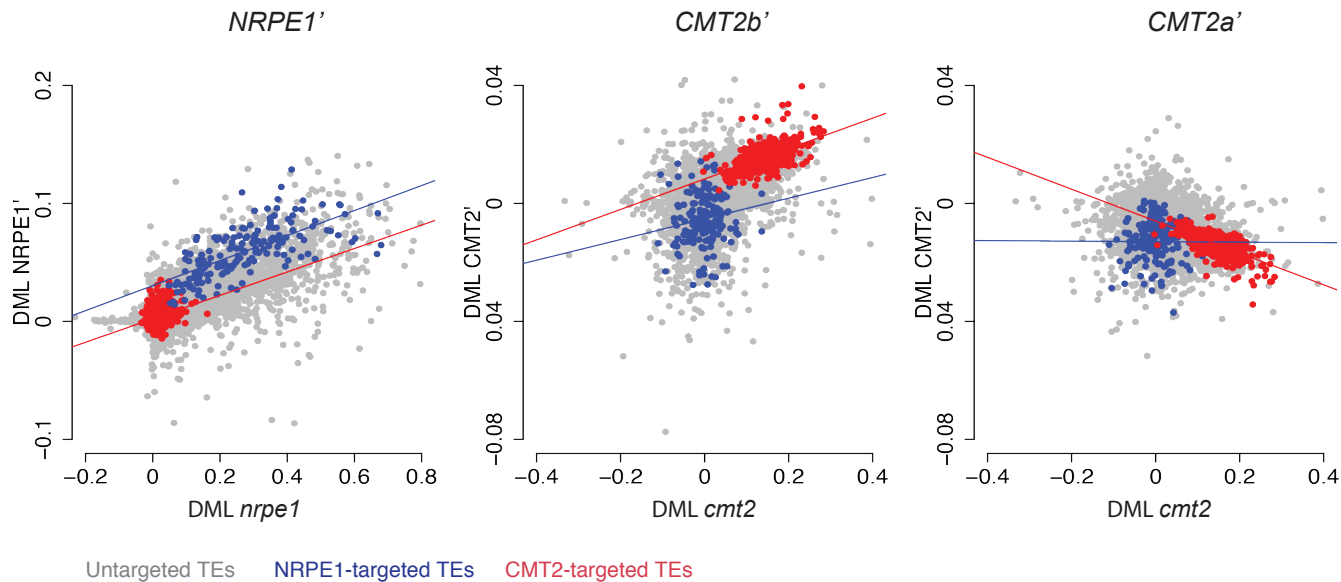

Supplement: S9 Fig — Correlation between molecular phenotypes of nrpe1-11 and cmt2 and the allelic effects on mCHH levels of TEs. NRPE1, CMT2-targeted, and untargeted TEs are shown in blue, red, and grey respectively based on GWAS results (-log10p-value>6 for NRPE1’ and CMT2b’). Regression lines are corresponding to NRPE1 and CMT2-targeted TEs. (PDF) [file pgen.1008492.s009.pdf]

S10 Fig

**A**

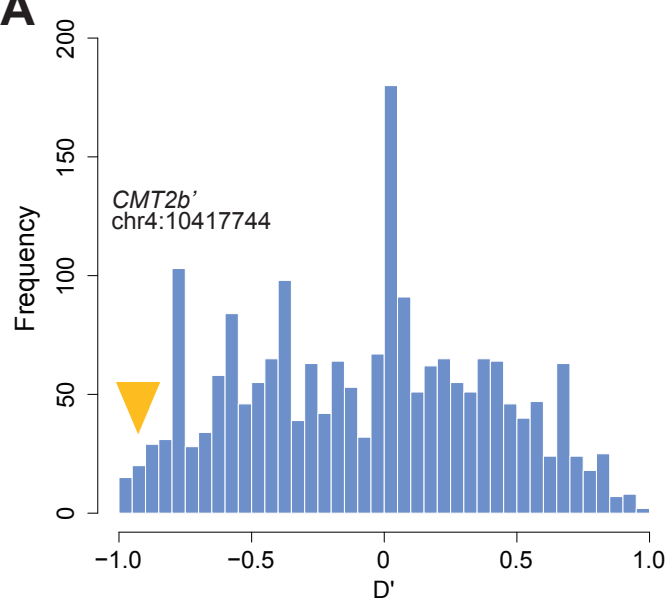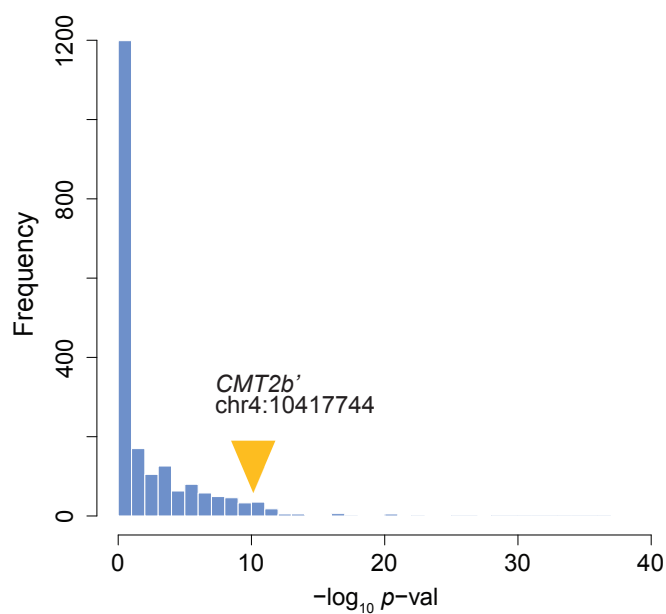

**B**

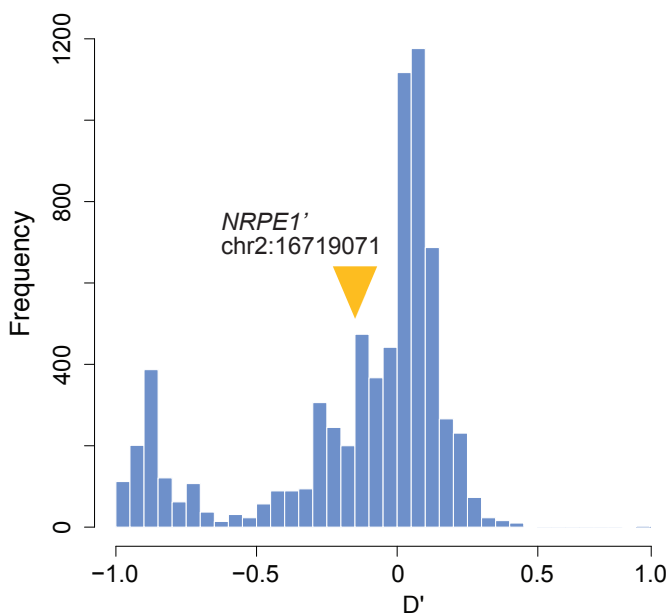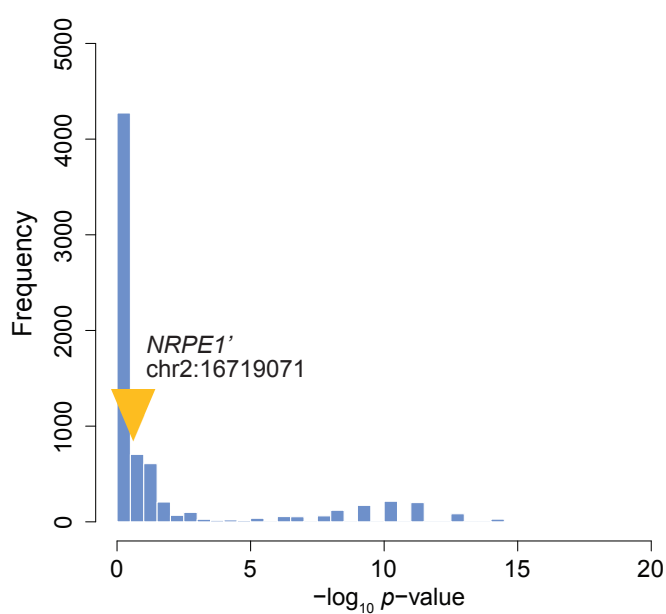

**C**

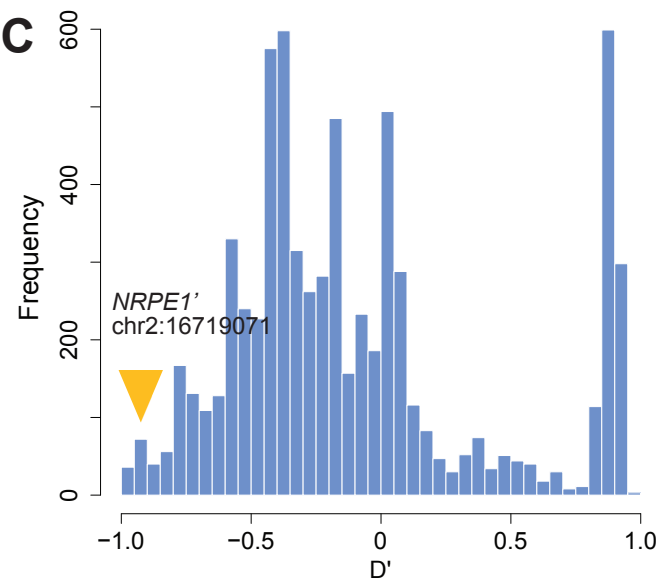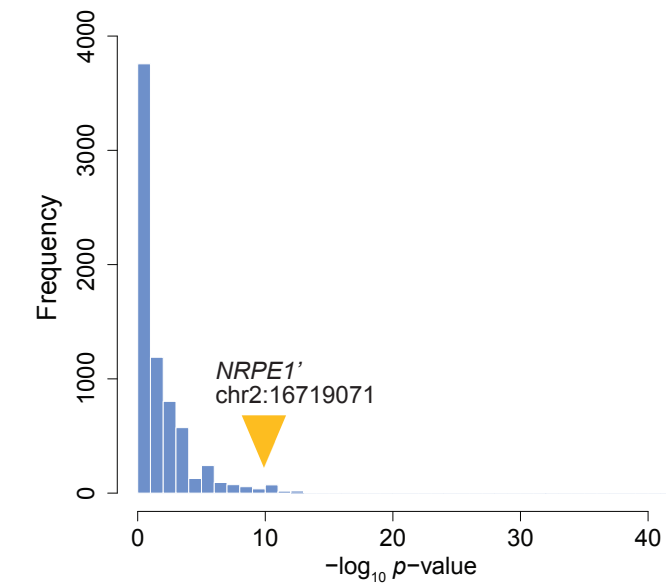

Supplement: S10 Fig — Plot A compares the value of D’ between NRPE1’ and CMT2b’ (orange arrow) to the distribution of D’ between NRPE1’ and genome-wide (unlinked) SNP of the same frequency as CMT2b’ on the left. The plot on the right shows the corresponding distribution of p-values calculated using Fisher’s Exact Test (one-sided). The empirical p-value of observing an association this strong is less 0.01. Plots B and C show the same, focusing on CMT2a’ and NRPE1’, and CMT2b’ and NRPE1’, respectively. (PDF) [file pgen.1008492.s010.pdf]

S11 Fig

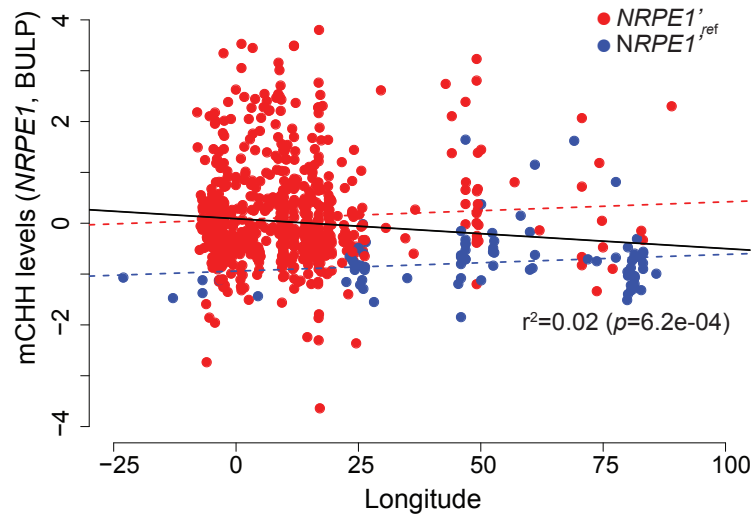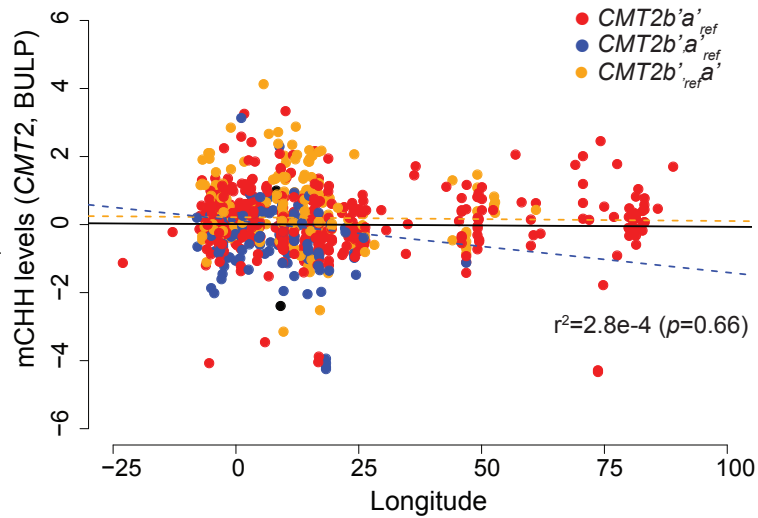

Supplement: S11 Fig — Plots show average mCHH levels of NRPE1- and CMT2-targeted TEs by taking into account population structure (BLUP) as a function of longitude. mCHH levels are averages of NRPE1’ and CMT2b’-targeted TEs. Colors of regression lines correspond to alleles; the black lines correspond to all lines. (PDF) [file pgen.1008492.s011.pdf]
