# Supplementary material for: Common alleles of CMT2 and NRPE1 are major determinants of CHH methylation variation in Arabidopsis thaliana
Source: PLoS Genet. 2019 Dec 30;15(12):e1008492. doi: 10.1371/journal.pgen.1008492 (PMC6953882; doi:10.1371/journal.pgen.1008492)
Supplement: S2 Table — (PDF) [file pgen.1008492.s013.pdf]

S2 Table. Top SNPs associated with mCHH variation (FDR20)

SNPs	MAC	X^2 TE families	X^2 TEs	*a priori* genes	SNP position
chr1:17895231	202	4140.11	54009.49	*AGO1*	Promoter
chr1:25391241	83	4571.31	64424.42		
chr1:27261944	62	3761.48	52716.43		
chr2:16719071	85	3598.75	51184.1	*NRPE1*	Exon
chr3:295927	168	3788.44	54896.41		
chr4:9595111	39	3857.09	55989.52		
chr4:10366178	282	3529.96	56425.33		
chr4:10405174	379	4718.38	82627.02	*CMT2*	5' upstream
chr4:10417744	145	4960.69	71492.15	*CMT2*	Exon
chr4:10421461	41	4245.6	65521.51	*CMT2*	3' downstream
chr4:10422486	190	6057.47	89703.87	*CMT2*	3' downstream
chr5:7041207	135	3715.7	54780.76		
chr5:7813881	484	4071.02	58694.87		
