# Supplementary material for: Common alleles of CMT2 and NRPE1 are major determinants of CHH methylation variation in Arabidopsis thaliana
Source: PLoS Genet. 2019 Dec 30;15(12):e1008492. doi: 10.1371/journal.pgen.1008492 (PMC6953882; doi:10.1371/journal.pgen.1008492)
Supplement: S3 Table — (PDF) [file pgen.1008492.s014.pdf]

S3 Table. Genetic effects on mCHH variation

SNPs	Alleles	% in total** (RdDM)	% in G	% in total (CMT2)	% in G
Total SNPs (Pseudo heritability)		55.2%		54.6%	
chr1:17895231[1]*	*AGO1*	5.6%	10.1%	1.0%	1.8%
chr1:25391241[2]		3.4%	6.2%	3.6%	6.6%
chr1:27261944[1]		2.2%	4.1%	3.1%	5.7%
chr2:16719071[1]	*NRPE1'*	6.6%	12.1%	0.8%	1.4%
chr3:295927[1]		0.0%	0.1%	0.8%	1.4%
chr4:10366178[2]		0.6%	1.1%	1.0%	1.8%
chr4:10405174[2]		0.7%	1.2%	2.0%	3.7%
chr4:10417744[2]	*CMT2b'*	0.0%	0.1%	3.6%	6.6%
chr4:10421461[2]		0.3%	0.5%	3.0%	5.5%
chr4:10422486[2]*	*CMT2a'*	0.9%	1.5%	4.3%	7.8%
chr4:9595111[1]		0.6%	1.1%	1.0%	1.8%
chr5:7041207[1]		3.9%	7.0%	4.6%	8.4%
chr5:7813881[1]		3.2%	5.9%	3.6%	6.6%
chr4:10417744,chr4:10422486	*CMT2a'*,*CMT2b'*	1.0%	1.9%	6.4%	11.7%
chr2:16719071,chr4:10417744, chr4:10422486	*NRPE1'*,*CMT2b'*, *CMT2a'*	7.3%	13.2%	7.4%	13.4%
RdDM related SNPs		18.3%	33.2%	12.6%	23.1%
CMT2 related SNPs		6.6%	12.1%	12.2%	22.3%
All SNPs		21.8%	39.6%	22.9%	41.6%

* is SNPs identified by Kawakatsu et al., 2016, ** % in total indicates how much phenotypic variation is explained by the alleles. [1] and [2] indicate SNPs associated with the RdDM and CMT2 pathway, respectively (Fig. 1)
