# Supplementary material for: Common alleles of CMT2 and NRPE1 are major determinants of CHH methylation variation in Arabidopsis thaliana
Source: PLoS Genet. 2019 Dec 30;15(12):e1008492. doi: 10.1371/journal.pgen.1008492 (PMC6953882; doi:10.1371/journal.pgen.1008492)
Supplement: S4 Table — (PDF) [file pgen.1008492.s015.pdf]

S4 Table. Compositions of TE superfamilies in Col-0 reference and it of common in the population (n=774)

	Reference (All TEs in Col-0)	Common
RC/Helitron	41.66%	48.90%
DNA/MuDR	17.41%	16.01%
LTR/Gypsy	13.45%	14.79%
DNA	5.89%	2.84%
LTR/Copia	5.73%	5.02%
LINE/L1	4.40%	3.21%
DNA/HAT	3.33%	2.66%
DNA/En-Spm	3.03%	3.31%
DNA/Harbinger	1.22%	1.07%
DNA/Pogo	1.11%	0.78%
RathE1_cons	0.69%	0.23%
DNA/Mariner	0.49%	0.12%
SINE	0.42%	0.29%
RathE3_cons	0.33%	0.18%
DNA/Tc1	0.31%	0.12%
LINE	0.26%	0.28%
RathE2_cons	0.24%	0.14%
null	0.05%	0.05%
