# Supplementary material for: Common alleles of CMT2 and NRPE1 are major determinants of CHH methylation variation in Arabidopsis thaliana
Source: PLoS Genet. 2019 Dec 30;15(12):e1008492. doi: 10.1371/journal.pgen.1008492 (PMC6953882; doi:10.1371/journal.pgen.1008492)
Supplement: Attachment — Submitted filename: Response to Reviewers.pdf [file pgen.1008492.s016.pdf]

Detailed response to the Reviewers

Thanks for the constructive comments. We agree with all of them and have explained our response in blue italics below. Changes are marked in red in the manuscript.

Reviewer #1

The study by Sasaki et al describes the identification of causal genes using GWAS for DNA methylation variation at transposon sequences within A. thaliana. Previous studies used bulk levels of DNA methylation within the 1135 epigenomes to identify putative natural variation at NRPE1 and CMT2. This study refines this approach by using mCHH at individual transposons, which significantly improve the resolution to pinpoint candidate natural variants. Numerous known and novel loci were identified and using previously published DNA methylome data from a collection of 86 A. thaliana mutants, they demonstrated the causal nature of the NRPE1 and CMT2 natural variants. These results further bolster this refined method for detection of natural variants controlling DNA methylation. Two examples of accessions possessing natural weak alleles of NRPE1 and CMT2 were identified at much lower frequency than expected by chance indicating selection against this combination. Lastly, integrating climate data there is an intriguing correlation between NRPE1 alleles and longitude.

Overall, this study nicely demonstrates the importance of the selection of phenotypic data for using GWAS to identify the causal nature of natural variants. The comments below are minor and are intended to improve this already strong manuscript.

1. The use of “de novo” methylation should be clarified. In the introduction it is stated that CMT2 is a de novo methyltransferase where as CMT3 is a maintenance methyltransferase. However, both recognize H3K9me2 to target DNA methylation. Given this mechanism is shared why is one considered maintenance and the other de novo. The type of methylation produce is irrelevant to this question. The use of CHH by the field to indicate de novo is overdue for a redefinition, as the Slotkin Lab has nicely shown, true de novo methylation is rare. Once true de novo methylation occurs at a region that previously was unmethylated all of the pathways, including RdDM ensue in maintenance methylation. This is important to this study due to the title and the opening introductory paragraph.

Thank you for the suggestion. We decided that “de novo methylation” is unnecessary as well as imprecise, and have replaced it with “CHH methylation”.

2. Line 13, CMT2 only binds H3K9me3 in vitro. As far as I know there is no known H3K9me3 in A. thaliana. And the one study that does report it is due to a antibody cross reaction with H3K36me3.

We simply followed the description of Stroud et al (2014), not realizing that this was controversial. However, since it is not important to our paper, we have rephrased line

13 as 'CMT2 preferentially methylates heterochromatic non-CG cytosines (Stroud et al., 2014; Zemach et al., 2014), ...', which is clearly true.

3. Figure 1—I think it is counterintuitive to display the methylation reduction the way that it is presented for drm1/drm2 and cmt2—it would be helpful if it was written under the key that you are showing percent reduction of methylation and what it is relative to.

We agreed, and have added the requested label to Figure 1.

4. Why is the pattern of correlation for differential CHH levels between cmt2/CMT2a' and cmt2/CMT2b' opposite?

Those are relative methylation levels compared to the reference allele. Since CMT2a' and CMT2b' show higher and lower mCHH levels respectively, the correlations with mCHH of cmt2 differ in direction. We added a sentence to the legend of Figure 3 noting this.

5. Figure 5—Why is cmt2a' / NRPE1ref methylation on cmt2 loci relatively high, but cmt2a' / NRPE1' and cmt2ref / NRPE1' show about the same level of methylation on these loci

Although NRPE1' clearly affects mCHH levels at the CMT2-targeted TEs negatively, it's still unclear how the CMT2 and NRPE1 alleles interact. Because of limited sample size (especially CMT2a'/NRPE1' lines), and confounding genetic background, we are unable to address this. To clarify which of the differences in Figure 5 we consider significant, we have added p-values to the text.

Reviewer #2

This manuscript presents a GWAS study of trans-regulation of Arabidopsis CHH methylation, based on 1001 Epigenomes data. It focuses on methylation of individual TEs and TE families, instead of genome averages (which were done by previous studies). The authors claim that this new analysis approach refined previous GWAS results and established causal relationship of the phenotype and CMT2 and NRPE1 alleles. In doing so, the authors also took advantage of the previously published methylome data from various Arabidopsis mutants and correlate with natural alleles. This analysis also identified several new associations; however, no further investigations were done. I think the results on CMT2 and NRPE1 are pretty convincing.

6. I have a minor comment on Figure 5A. It is obvious that CMT2b' is insignificant on mCHH levels of RdDM targets, while with NRPE1ref (orange and green lines basically overlap in Figure 5A top left panel and the authors also provided p value). However, it seems that CMT2b' has lower methylation than CMT2ref or CMT2a', while in NRPE1' background (dark blue line vs. light blue and magenta lines). Moreover, although the authors say cmt2 knock-out is similar to CMT2b', Figure 5A top right panel suggests that there is clear difference between reference and cmt2. In order to claim statistical significance or insignificance, p values of these pair-wise comparisons should be provided.

We agree, but since only two lines carry CMT2b' / NRPE1', we cannot assess the significance. As noted in the response to Reviewer #1, we have added p-values to the text to clarify which differences we consider important.

We also agree that the cmt2 loss-of-function allele shows a weak but clear reduction of mCHH levels at RdDM-targeted TEs. However, our point is that there is qualitative agreement with our GWAS result in that CMT2 is not a major determinant of mCHH levels at RdDM-targeted TEs (unlike for CMT2-targeted TEs). We rephrased the sentence to make this clearer.

7. In the case of AGO1, based on Figure S7, the effect of ago1 mutant on methylation is rather small. On line 125, did the authors mean to say the allelic effects are too small or the mutant effects are too small? The problem with this interpretation is that the ago1 mutant methylation data from Stroud et al Cell 2013 (which the authors of this study used according to Materials and Methods) is obtained from ago1-27, which is a hypomorphic, not a loss-of-function, allele (according to Morel et al Plant Cell 2002). True null or loss-of-function alleles of ago1 are lethal. In additional, there could be functional redundancy of other Agos that can compensate for reduction of ago1's function in DNA methylation. Therefore, I would suggest the authors revise the part on ago1, or it will be misleading.

Thanks for pointing this out. We agree, and amended the description on Lines 124-125 to note this possibility.
